# Food, power and agency: revealing local post-harvest fisheries practices to improve food access from small-scale fisheries in coastal Kenya

**DOI:** 10.1007/s40152-025-00402-7

**Published:** 2025-01-29

**Authors:** Antonio Allegretti, Johnstone O. Omukoto, Christina C. Hicks

**Affiliations:** 1 Lancaster Environment Centre, Lancaster University, Bailrigg, LA1 4YQ UK; 2 Kenya Marine and Fisheries Research Institute (KMFRI), Mombasa, Kenya

**Keywords:** Food, Power, Agency, Ethnography, Social sciences, Kenya

## Abstract

This article proposes the case of Kenyan coastal fisheries as a potentially crucial reservoir of food-related benefits for the marginalised and those living in poverty, but where a food-centred lens or approach is seldom mainstreamed in local and national governance. Borrowing insights from post-structuralist marine social sciences, this article presents an ethnographic account of grassroots practices in-the-making such as handling, sorting, and allocating fish once caught, and how these practices lead to local categorisations and classifications of fish. This sort of evidence and knowledge around local categorisations and classifications of fish spotlights the importance of considering the post-harvest sector (as opposed to the activity of fishing alone), that is, how the use of catch determines access through micro relations of power and agency. Through the analysis of two different locations of Watamu and Shimoni in terms of the fisheries economy and overall development, the analysis of these categories and classifications highlights the necessity to account for a fairer access and distribution rather than solely production (of fish) that is overly market-oriented.

## Introduction

Food insecurity and malnutrition are persistent and worsening (FAO et al. 2023). In 2022 nearly 10% of the world’s population was hungry, nearly 30% affected by food insecurity, and 42% unable to afford a healthy diet; a situation that is worse now than when the SDG goal of zero hunger was set in 2015 (FAO et al. 2023). These challenges are particularly acute in rural areas of the Global South, including sub-Saharan Africa. Yet, in these contexts, fish-based food systems provide affordable and nutritious food (Omukoto et al. 2024a, b; Robinson et al. 2022). Fisheries can therefore play an important role in supporting food and nutrition security, if governed to ensure sustainability and to enable access for those most in need (Allegretti & Hicks 2023; Bavinck et al 2023).

To strengthen the link between fisheries management and food access, in this article, we focus on the post-harvest phase of Kenyan reef fisheries, and its role in supporting access to fish as a valuable source of nutrient-rich food for the most marginalised communities (Darling 2014; Galligan & McClanahan 2024). We delve into how the catch is handled and managed through micro negotiations, and how localised power relations involving the catch shape people’s agency in accessing fish, particularly for those in poverty and those most vulnerable. Through the prism of localised power relations, and informed by post-structuralist marine social science (Mather et al. 2017), we illustrate a variety of practices that are not generally accounted for in formal fisheries governance (Allegretti & Hicks 2022; Basurto et al. 2020); local categorisations and classifications of fish emerge as a result of these practices, with effects on how and where fish enter markets and trade.

Fisheries management and policy are generally concerned with the harvesting phase, falling under the remit of environmentally focused research and government departments that examine what is caught, by whom, and where (Basurto et al. 2020). Scientific categories of size and species underpin most reef fisheries management practices in Kenya (Hicks & McClanahan 2012); gear-based management sets mesh-size limits that allow larger individuals to escape (Hicks & McClanahan 2012) and prohibit the use of destructive gears that indiscriminately target all species and sizes of fish (Mbaru et al. 2021; Mbaru & McClanahan 2013). Although gear-based management, has slowed or reversed long term declines in fishers' catches and incomes (McClanahan 2021; Mclanahan et al. 2008), community level food-related objectives remain mostly unaddressed (Odoli et al. 2019). If Kenya’s fisheries are to be governed such that they can effectively support food security, the focus needs to expand beyond activities at sea, and particularly to how power determines people’s agency to acquire fish, in the post-harvest phase. With current Blue Economy agendas increasingly taking centr stage in coastal development in Kenya with the expansion of investment and activities (e.g. ports) (Thoya et al. 2022), introducing food-related concerns into fisheries management becomes critical to balance multiple needs and strengthen bottom-up participation for equitable governance (Thoya et al. 2022).

Our analysis of power and agency in the post-harvest sector builds on current food security understanding and discourse. These have been subject to a significant transformation in terms of ‘rescaling’ from a focus on states, informed by the rather technical ‘more-food-equals-more-food-secure nations’ equation, to the conditions that enable or constrain access to food at the local level (Clapp et al. 2022; Hopma & Woods 2014; Sonnino et al. 2016; Wald & Hill 2016). Rooted in Sen’s understanding of agency as a ‘person’s ability to act on behalf of what he or she values’ (Alkire 2008), the ability of groups and individuals to make choices to achieve valued food-related outcomes has become more central in food security analyses and high-level policy processes (Clapp et al. 2022; HLPE 2020). This transformation has led to a focus on how power, locally, affects dietary patterns, choices, and outcomes (Sinharoy et al. 2019).

The shifting paradigm around power in food security builds on an already rich debate in social science theories around the *relational* dimension of power and agency (Butler 2010; Reed & Weinman 2019; Zaaiman 2021). Marine social sciences have paid increasing attention to questions of power and agency with analyses of local practices and local categorisations through ‘deconstruction’ of formal categories and technologies of governance (Mather et al. 2017). Here, we depart from the ‘deconstruction’ of science-based formal management categories applied in the ‘harvest’ phases, commonly focused on size and species, to analyse the local categories which are produced through local practices in the post-harvest phase.

The focus on the post-harvest phase of fisheries will inform the understanding of how ‘territorial markets’ (Bavinck et al. 2023) play out in the context of coastal Kenya. These are crucial for food security in the Global South, and have recently branched out into more complex and longer value chains with a more varied landscape of actors and development (Bavinck et al. 2023). Yet, they retain a markedly ‘local’ character that differentiates them from global commercial seafood trade (Asche et al. 2015), with the consequent disconnection from the logic of market-based interventions that in the last two decades have gained prominence for achieving global sustainability objectives (Kittinger et al. 2021; Murphy et al. 2021). This gap calls for detailed analyses of local practices to better understand growing power imbalances within territorial markets, and how these affect the agencies of their key actors, and consequently, access to affordable fish by local consumers (Bavinck et al. 2023).

## Deconstruction, performativity, and power: using a post-structuralist marine social sciences approach to explore practices, classifications, and categorisations

Post-structuralism in social theory in the 1960s and 1970s started as a counter-hegemonic reaction to an over reliance on categories or ‘structures’ used to present reality as an ontological given (Woodward et al. 2009). Post-structuralism in social sciences, particularly associated with French thinkers such as Foucault, Bourdieu, De Certeau, questioned a priori forms of knowledge such as narratives, representations, and text. It introduced a set of new ideas aimed at unearthing what remains concealed in these narratives and representations such as power, practices or *performances*, agency, making ‘deconstruction’ the core of its agenda (Woodward et al. 2009).

Post-structuralist approaches to fisheries and ocean research have been applied to deconstruct formal management and governance tools, categories and technologies, from Individual Transferable Quota to stock assessments (Mather et al. 2017). Researchers have illustrated the ways in which these approaches fail to consider the importance of local agencies and knowledge (Mather et al. 2017). From the analysis of networks created by Indian fish trawlers striving to (re)claim their space in formal allocation of ocean territory (Stephen 2015), to the production of local categorisations and classifications of fish by anglers in UK that defied scientific traits of size and species (Bear & Eden 2011), post-structuralist marine social scientists have brought to the fore the variety of *practices* that local actors employ to (re)claim and exercise their agencies, locally produce ‘alternative’ orders, and engage with technologies of governance (Johnsen 2017).

Here, we employ a post-structuralist approach to the analysis of the practices of handling fish to spotlight the importance of the post-harvest sector as a critical space in which people’s agency to access fish as food is determined. Departing from the three pillars of deconstruction, performativity, and power laid out by Mather et al. (2017), we *deconstruct* formal management categories based on fish size and species. We focus on local and informal management in the post-harvesting phase to uncover how it relies on local categories that emerge out of *performances* (sorting, classifying, allocating). Understanding how local categories come about helps uncover how fish shift between different regimes of value for example, between fish as a tradeable commodity and fish-as-gift for local consumption (Lien & Law 2011) and enter different market channels affecting access to fish by the local population. In looking at these practices, we analyse the micro-politics and relations of *power* among the parties involved, such as fishers, dealers, and traders which contribute to the fluidity of local categories and determine the agency to acquire fish for food for those who need it most.

## Materials and methods

### Study area

Coastal small-scale fisheries in Kenya are diverse and complex due to the often informal nature of economic activities, the use of multiple gears, targeting of multiple species, the seasonal variability of the fishery, and presence of migrant fishers (Fulanda et al. 2009; Wanyonyi et al. 2016). Along the coast, it is common to find a mixture of different species and sizes of fish on the market, with small fishes comprising both mature small pelagic fishes such as sardines, anchovies, and clupeids, as well as immature small demersal coral reef fishes such as rabbitfish, snappers, scavengers, and parrotfishes.^1^

**Map 1:**
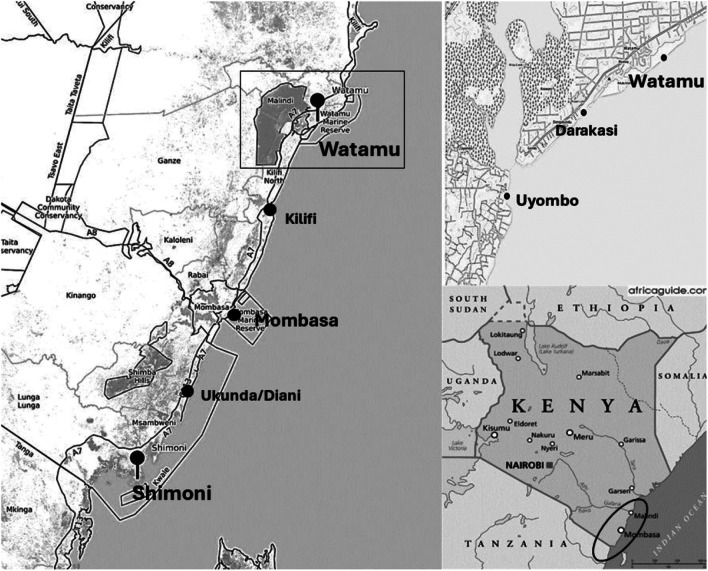
Coastal Kenya with fieldwork locations. Authors’ own map produced with https://caltopo.com/

Here, we present a comparison between two major fishing centres in coastal Kenya, Shimoni in the south coast and Watamu in the north (Map 1). The two locations present some socio-economic differences – less populated with more subsistence livelihood activities in the first, and more populated with more pronounced tourism development in the second (Table 1). There is a mix of fisheries operating out of the two sites. These include a mix of mangrove-seagrass-coral reef-associated fisheries and more distant pelagic fisheries. These fisheries are diverse in terms of crafts, gears, species, and degrees of market integration. The smaller-scale artisanal fisheries tend to operate within the lagoon, close to shore (McClanahan 2010), supplying local markets with seagrass and reef-associated fish, including rabbit fishes, scavengers/emperors, parrot fish, and snappers (McClanahan 2010). The larger more developed fisheries tend to venture beyond the reef, into open waters using more advanced gear, and supplying larger market centres with bigger pelagic families, including carangids, mackerels and tuna (McClanahan 2010).

**Table 1:** Socio-economic features of the two sites

Characteristics	Shimoni	Watamu
Geographic position *	4°38′49.79"S; 39°22′49.49"E	3°21′12.85"S; 40°01′17.97"E
Total population estimates **	6,520	12,286
Number of households **	1,618	3,537
Main livelihood activities	Fishing, subsistence farming and livestock keeping, some tourism activities and other formal/informal employment	Fishing, food-related businesses (shops and restaurants), marked tourism activities (employment in hotels, sales of art crafts, tour guiding etc..)
Type of fisheries	Widespread use of artisanal gears such as basket traps, fence traps, hand lines, gill nets, cast nets, and long lines. Overall, lower level of capital investment	More developed gears such as trolling, used in open waters beyond the reef. Overall, higher level of capital investment

*Estimation from Google Earth Pro

**As per the 2019 National Census for the sublocation level

While the two types of fisheries co-exist in most locations (including the two locations considered here), some differences do exist between Shimoni and Watamu. Artisanal gears, sometimes handmade, such as basket traps, fence traps, hand lines, gill nets, cast nets, and long lines are more popular in smaller landing sites with lower levels of capital investment such as in Shimoni. These landing sites are used by Kenyan fishermen throughout the year and migrant fishermen from Tanzania during the ‘high’ dry Northeast Monsoon (Ochiewo 2004). More developed gears such as trolling, used in open waters beyond the reef, are more popular at larger landing sites such as Watamu which are characterised by higher levels of capital investment, and where there are widespread high-end recreational fishing activities (Kadagi et al. 2020) (Table 1).

### Ethnography of power relations

Ethnography is the main method utilised for the research reported in this article. Ethnography is closely interlinked with the post-structuralist agenda of analytical deconstruction of representations, discourses, and categories (Britzman 1995), and an important analytical tool of social science disciplines such as anthropology and cultural geography to analyse power and performativity (Spry 2006). Ethnography has traditionally been defined by the practice of *participant observation* in anthropology with prolonged periods of time spent by the ethnographer to develop rapport and relationships of trust with the research subject (De Walt & De Walt 2011).

In marine social sciences, ethnography is increasingly being utilised to bring out local power dynamics (Allegretti 2019; Lyons et al. 2016), networks and relations (Allegretti 2019; Dobeson 2018), local perspectives, priorities and knowledge (Fabinyi et al. 2015), including in the post-harvesting phase (Lavoie et al. 2019), and to explore fluidity of boundaries in fish markets and trade (Dobeson 2018). These are instances of local knowledge that can only be unearthed through intensive observation and participation of the researcher in local practices (Fienup-Riordan et al. 2013) that often remain invisible in formal management (Lavoie et al. 2019).

**Fig. 1:**
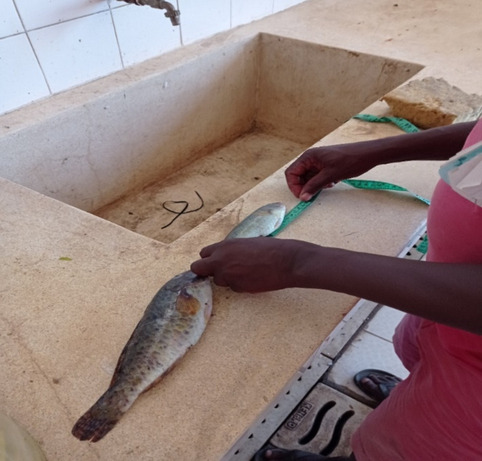
Data collector at Uyombo Beach Management Unit collecting size-based data of the catch

Here, we utilise ethnography^2^ to bring out local categorisations that emerge out of local values and knowledge, process, relations, and performances. We used a combination of participant observation and interviews, all conducted by the lead author in Swahili, the national language of Kenya utilised by most people on the coast, with notes taken also by the lead author but in English. I (the lead author) spent a period of around three months (January to March 2022) observing practices of handling fish after harvest. I observed a wide range of practices surrounding the use of the catch such as sorting, categorising, allocating. These often are *performed* at or in proximity of the Beach Management Units (BMUs) and other major landing sites, i.e. the nerve centres of fisheries local management (Allegretti 2019; Nunan et al. 2015) where different actors interact, determining different trading patterns. The ethnography was conducted for the most part at the BMUs of Shimoni, Watamu and Uyombo, a satellite of Watamu, and at the landing site of Darakasi in Watamu (Fig 1). While in Shimoni, two shorter visits were made to the island of Wasini, off Shimoni, a 15-min boat trip.

Having gained trust and permission to conduct fieldwork at the BMUs from the local authorities (having already obtained research clearance from NACOSTI^3^), I built rapport with some key informants among BMU managers, traders, both men and women, and fishermen, and started closely observing the practices of handling fish. In time, I was increasingly able to ask people to explain how practices of sorting, categorizing, and allocating fish are performed in-the-making. This enabled me to delve into the factors that determine value attributed to different fish, and the micro-politics around handling fish, that is, different levels of *power* that determine how fish is sorted and allocated to different market channels. I utilised note-taking as a recording technique while observing the practices and asking questions in-the-making, and set time at the end of the day, mostly in the evenings, to write down critical reflections for data analysis; all notes recorded were dated and notebooks numbered to facilitate the subsequent data preparation and analysis (see below).

After around one month of participant observation, having gained an understanding of the practices of handling fish, I conducted in-depth interviews with selected individuals (having gained informed consent) to further delve into how and why different actors (can) exercise higher or lower levels of power over others. No given statistical principle or tool was utilised to determine the number of interviews as representative sample of the general population; rather, I interviewed key informants until I gained an appropriate understanding of the questions that enabled me to fulfil the research objectives. This approach led me to conduct twenty interviews (ten at each location, Shimoni and Watamu) with key informants among fishermen, members of BMUs, fish dealers, and fish traders. Dealers are mostly males, and do not sell fish directly to consumers but rather purchase fish catches to resell to either other dealers or to traders. Traders sell fish to consumers in established and permanent shops or kiosks, or mobile street stalls, obtaining their supplies either from dealers or directly from fishermen. While shop and kiosk owners can be either men or women, those selling in mobile street stalls are local women who process fish (e.g. frying) before selling it and are known as Mama Karanga (from the Swahili verb *ku-kaanga*, to fry). Some dealers own fish shops, hence they can operate as retail traders as well.

Male dealers (from now on ‘dealers’), traders, and Mama Karanga interviewed were long-term residents of the communities where they operated; however, some dealers visited the communities where they got the supplies (including Shimoni and Watamu) while residing in other parts of the coastal region (urban centres such as Ukunda, Mombasa, Kilifi and Malindi). Overall, dealers are more likely to have external links, for instance with fish shops along the coast or in Nairobi, whereas traders and Mama Karanga mostly operate locally only. Some dealers act as intermediaries in exports of small pelagic species to Congo or China through fish exporting companies such as Huawen Food Kenya Ltd. The focus on power relations during the interviews led to discussions around trading and market networks, the overall trading activity in terms of size of catch, and people’s own preferences around fish.

Following the ethnographic fieldwork, at the end of the three months of research, a validation stakeholder workshop was organised with representatives from different categories of stakeholders who had taken part in the research, and further discussion occurred around the institutional arrangements that contribute to determine access to fish by more marginalised actors of the fisheries value chain. At the end of the fieldwork period, all notebooks where ethnographic observations and data from interviews where recorded were manually reviewed, screened, and information categorised by the first author in consultation with the other authors according to recurring themes, using references to dates and page numbers in the notebooks, and to produce a narrative around the practices analysed, how these create local categories of fish, and the related determinants of power and agency.

## Findings and discussion

### Sorting, categorising, allocating: making the local grading system

#### Production of fish categories through performance

Three classifications of fish emerged during the ethnographic observation: 1. ‘small’; 2. ‘large’, and 3. *kitoweo* (Table 2.). ‘Small’ fish include mostly reef associated fish (e.g. parrotfishes – “pono”, emperor/scavengers – “changu”, rabbitfishes – “tafi") of different sizes but also some long-bodied eels, small barracudas, needle fishes, halfbeaks, and small pelagic species including sardines and anchovies. ‘Large’ fish include mostly large pelagic fishes (e.g. tuna) that are often caught with better quality gear in semi-industrialised fisheries, but also reef associated fish of a larger size. *Kitoweo* is a Swahili word that refers to fish that is outside the commoditisation sphere and is used as gift for friends and family and for household consumption (Wamukota & McClanahan 2017). What fish goes in what basket is not a simple matter of blunt size, as for instance a small sized fish can make it into the ‘large’ fish basket (or vice versa), nor is the commodity-gift boundary fixed, as for instance *kitoweo* can be traded through market channels. Instead, and as described below, a host of elements and dynamics, including of power, contribute to determine, through practices and performances, what fish is categorised as ‘small’ or ‘large’.

**Table 2:** Local categories of fish

Fish category	Fish types (species)	Geographies of fish categories		Specific local traits
‘Small’	Reef associated fish (parrotfish, scavenger, rabbitfish), long-bodied fish (eels), small pelagic (anchovies, sardines)	**Shimoni**	**Watamu**	Suitable for local processing (by Mama Karanga) Referred to as *changu* (Emperor/scavenger fish) beyond scientific taxonomy
		Sorted and arranged in bundles at BMU for local markets alongside ‘large’ fish	Weighed separately for local markets (result of marginalisation)	
‘Large’	Large pelagic (e.g. tuna)	Sorted at BMU, allocated to dealers for higher end markets through negotiations of boundaries with ‘small’ fish	Weighed at BMU for higher end markets for hotels	Require higher cooking skills (professional chefs)
*Kitoweo*	Mixed, mostly from ‘small’ basket	Mostly taken to households for consumption or extended as gift	Pulled out of bundles of ‘large’ fish at BMU, and often marketed *as kitoweo*	Referred to as *mboga*

Fish is first emptied into buckets or containers of different types and sizes (buckets, large bags, boxes), either inside or outside the BMU premises. Buckets, bags, and boxes are then emptied and the fish poured onto the counters used to handle fish inside the BMU (e.g. for measuring, counting, removing fish guts, scaling, and washing) (Fig. 1) and the data on size and counts are recorded by species or taxa on notebooks carried around by the data collectors. The data collectors are BMU members with active or prior positions (chairs, secretary, etc..) who normally volunteer for the activity or, on some occasions, county fisheries officers and research scientists who carry out catch assessment surveys during designated periods (such as bi-weekly, or quarterly) over a year. Data collectors then begin counting the fish according to size; one fish of X size (starting with smaller sized fish and getting progressively larger) is chosen as the unit of measurement and all other fishes of approximately the same size are sorted in one corner before the same happens with another size-based category of fish (Fig. 1).

Data collection is done while more catches are brought in and in turn sorted according to size by the fishermen and dealers themselves. Following the collection of data, fish is weighed using the scales provided at the landing sites by the BMU or the dealers/traders. Weighing is done to the nearest 250 g for large fish and in bundled quantities (piles, basins, or buckets of known or estimated weights) for small fishes that include sardines and anchovies.

**Fig. 2:**
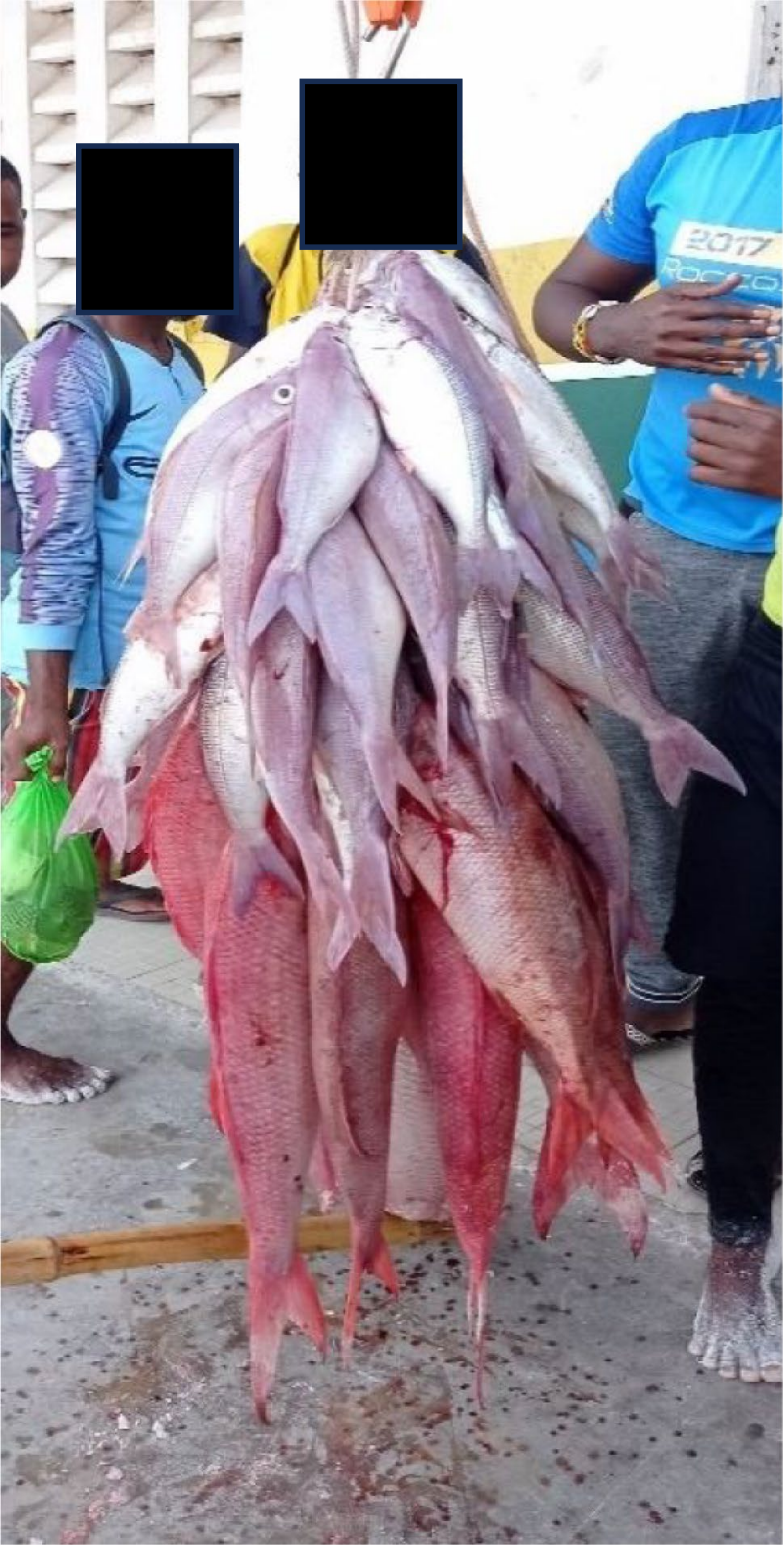
Weighing fish at the Watamu Beach Management Unit

Local practices of sorting, categorising, allocating, however, do not correspond to the size-based measurements that are carried out when fishermen report to the BMU with their catches. Instead, they differ from one location to another. In Watamu fish categorisation starts while fishers are still at sea where they assemble large bundles (Fig. 2) with bigger sized fish at the bottom of the bundle, and progressively smaller fish on the outside and top of the bundle. These smaller fishes are normally placed in such a way that they can be easily pulled out as *kitoweo*. At docking time, normally twice a day (early morning and evening), people gather around the BMU premises creating a generally frenetic atmosphere in which it is difficult to determine the function and role different individuals play, between weighing, carrying catches, dealing fish, or simply watching. In this atmosphere, the fish bundles are carried by casual local workers after landing on the shore immediately adjacent to the location outside the BMU gate where the scale is placed.

Following the allocation of the catch to dealers, it is sorted and weighed. This activity is more straightforward where prior agreements exist on price per kilogram, as for instance when there are patron-client relationships; patron-client relationships include a range of arrangements such as fishermen supplying dealers with fish in exchange for fishing inputs (such as fishing equipment, boats or boat engines, and fuel), or flexible loans in time of need and/or access to markets. Where such agreements are not in place, allocation of the catch is the result of in situ negotiations around price but also, as will be seen, size of fish. These negotiations occur when fishermen sell to freelance dealers or to Mama Karanga. The handover of the catch is a crucial step that in large part determines what fish enters what market channels, with important implications for what and how much fish are available for local access versus that entering larger markets mostly along the coast, but also in Nairobi or outside of the country (e.g. Congo, China).

**Fig. 3:**
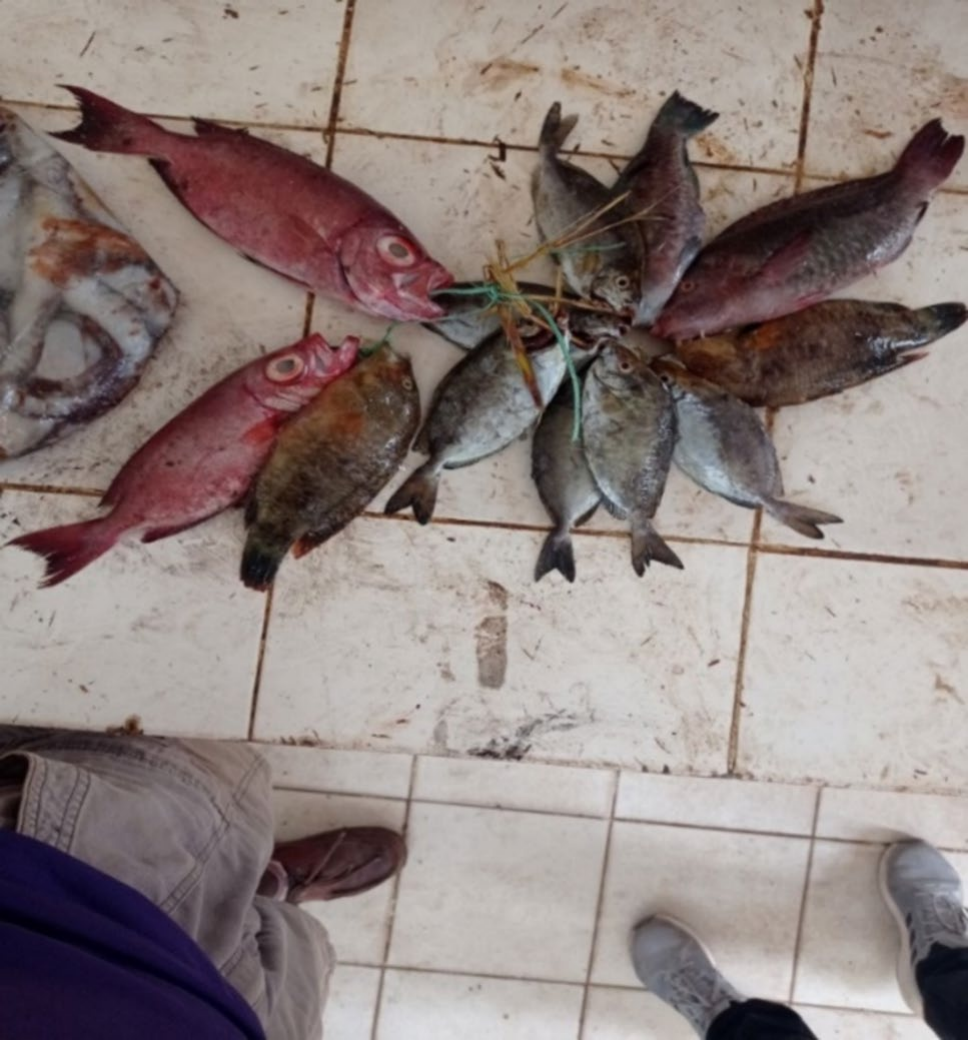
A bundle of ‘small’ fish at Shimoni Beach Management

In Shimoni, ‘large’ fish are generally placed in large baskets that are taken to distant markets; whereas, ‘small’ fish are arranged, at the BMU, into small bundles, that rarely exceed ten fish, attached by a small rope woven through the gills of the fish (Fig. 3). These bundles are put together for small-scale local traders selling fish in shops or kiosks that service local markets or local eateries. Bundles of ‘small’' fish in Shimoni are also used as *kitoweo* and taken home by fishers for consumption.

#### Fish qualities, traits, and local value: problematising fish categories

The practices of sorting and allocating fish hinge on a number of complex traits and qualities of fish that are locally determined, rather than dependent on the scientific categories of size and species utilised in the management of the ‘harvesting’ phase. In the end, these practices create blurred boundaries between the different local categories and preferences for fish. For instance, Mama Karanga prefer ‘small’ fish that can be easily processed whole, maintaining intact their physical properties when deep-fried in oil. The same species of fish but of a larger size can also be earmarked as ‘small’, despite the large size—they are chopped into pieces before being fried, retaining their qualities through the processing. This crucial trait of ‘small’ fish valued for its ease of processing by local women beyond bare size was highlighted on several occasions; the opposite was also highlighted by one dealer in Watamu, that is, smaller fish being earmarked as ‘large’—he picked up a fish and argued:

“See this fish? [picking up a small fish] Sometimes dealers add this to the buckets that Mama Karanga bring here because they think they will like it for being small. But when Mama Karanga see that, they would shout and say ‘take it out!’ because when you fry it, its skin comes off and the whole fish is ruined. This needs some real professional cooking of the kind chefs in hotels here can do, not our mamas. So we just add it to the lots of large fish that are taken to Mombasa or Nairobi, or hotels where the professional chefs know how to cook it”.

Much like size, local categorisations of fish intersect with, and in some cases override, scientific species-based taxonomies. Much of the fish that is categorised as ‘small’ and allocated to local markets is often labelled as *changu*, the Swahili name for Lethrinidae species, commonly associated with coral reef ecosystems. A size-based differentiating between ‘small’ and ‘large’ *changu* is not a clear-cut process; according to the estimation of one fisherman interviewed, one kg of *changu* is made up of more than 9–10 individual fishes of different species, and it is categorised as ‘small’ to stay within local market networks rather than being taken to higher-end markets.

Ambiguous properties exist around *kitoweo* as well. This is fish for household consumption (Wamukota & McClanahan 2017), otherwise referred to, more generally, as *mboga*, that is, any complement to the maize-based staple of *ugali* (thick maize porridge). *Kitoweo* fulfills the function of a household gift for consumption and often is made to belong to the same species-based basket of *changu*. Both in Shimoni and Watamu, one could easily see fishermen, dealers, as well as ordinary villagers carry small bags with a modest sized portion of fish, made of a single larger fish or a group of 4 to 5 fishes for the household. Asked about what the fish is for, most would say that the fish is a ‘gift’, ‘for my sister’, ‘for my mother’, ‘for home’.

Just like the ‘small’ and ‘large’ categories, the category of *kitoweo* is loosely formed through negotiations of boundaries between what is and what is not to be allocated to household consumption. These are boundaries that correspond to market and non-market spheres of commoditisation, and are, like other categories, blurred. Fish categorised as *kitoweo* can be either earmarked before fish is sorted, for instance when fishermen take some of the fish for their household before handing over the catch to the dealer, or can be marketed *as kitoweo* by the dealer. This second case emerges as blurred boundaries between the categories of ‘small’ and *kitoweo* – most dealers would refer to the fish they collect at the BMU for resale as *kitoweo* for household consumption.

Having defined how local categorisations of fish are created, the next two sections build on the local categorisation system organised around the three categories of ‘small’ fish, ‘large’ fish, and *kitoweo*, to explore how micro-politics and relations of power among the parties involved contribute to the fluidity of these categories. These will be analysed through power and agency as analytical keys that explore the conditions that determine access to fish by local populations in Shimoni and Watamu.

### ‘Small’ fish, ‘large’ fish in Shimoni

#### Dealers, fishers, and bargaining power

Limited post-harvest facilities and the use of gears (e.g. basket traps, handlines, gill nets) that require lower capital inputs distinguish Shimoni fisheries from fisheries with higher capital investment (such as in Watamu), but these fisheries with low level of capitalisation can have positive effects. The most obvious positive implication of limited storage facilities is that fish is likely to enter local market networks for immediate consumption by local people, reaching those with limited purchasing power including poorer and more vulnerable individuals in communities, rather than building larger stocks that can be sold in bulk to more distant markets or to higher end hotels and resorts (as is the case in Watamu). Different channels exist for local people to access fish, such as fishermen taking fish directly to their households for family consumption, extending fish as gift (*kitoweo*) to family members and friends, as well as through local markets, at the BMU, or through the commercial mediation of Mama Karanga (Cartmill et al. 2022).

Given the overall small-scale nature of the economy, with limited market networks and widespread artisanal gears, in Shimoni, most dealers and traders tend to be positioned on the lower end along the low-to-high-capital-based continuum (Kimani et al. 2020). Dealers and traders are more involved in local retail and processing (especially women), rather than coordinating distribution to access larger markets and high end consumers; activities associated with higher-capital dealers and traders (Kimani et al. 2020). In Kenya, as elsewhere, relationships between fishermen and the whole host of dealers and traders who act as intermediaries between the harvesting phase (fishing) and the end consumers have a bearing on access, (in)equality, and the distribution of fish (Miñarro et al. 2016; Wamukota et al. 2014). In Shimoni, the trading activity is smaller in scale, which in turn entails more equal power relations with fishermen and has positive implications for access to fish by local communities.

Based on interviews with traders and BMU executives in Shimoni, it was estimated that a maximum of 50–60 kg per day of fish are traded by an average capital-endowed local dealer. There was consensus that the revenues generated from these quantities of fish can create a small margin for reinvestment beyond next-day trading (i.e. money necessary to purchase fish for next-day sales), leaving little room for expansion beyond local markets. As a dealer in Shimoni explained, while occasional deals occur between dealers in Shimoni and other higher-end dealers or traders in bigger locations (Diani, Mombasa), sustaining long haul trading necessitates capital for long-term deals, often on a monthly basis. This means a trader in Shimoni would need a month’s worth of capital to purchase fish to supply ‘next-of-chain’ dealers or traders until pay day—capital that most dealers in Shimoni do not have.

Competition among dealers occurs around the purchasing price offered by individual dealers to fishermen. Price-based competition among dealers results in fishermen having more bargaining power when dealing with these freelance dealers as opposed to when they are involved in patron-client relationships that are often unequal or exploitative (Ferrol-Schulte et al. 2014). More equal bargaining power between dealers and fishermen results in more volatile partnerships between the two actors in the chain; the dealers interviewed estimated that a partnership between a fisherman and a dealer can last anywhere between one single transaction and a few months, and are almost never exclusive, meaning that fishermen continue to sell to other dealers when they can fetch a better price. This happens even when fishermen are tied to exclusive patron-client relationships which often cause arguments that have to be handled by the BMU executives (Interview with BMU member, Shimoni).

#### Performance, agency, access

More equal bargaining power between fishermen and dealers play out during the performances of sorting, categorising, and allocating fish which ultimately shape markets of ‘small’ and ‘large’ fish. Small and large fish fetch different prices for fishermen; fish is either sorted into ‘small’ and ‘large’ by the fishermen and then sold to different dealers and traders, or a dealer can buy the whole catch and sort fish (into ‘small’ and’large’) together with the fisherman to determine the final price. ‘Small’ fish are normally purchased by Mama Karanga at the BMU, while ‘large’ fish are immediately transported to close-by locations to supply local shops, restaurants, or occasionally to larger markets such as Diani. One dealer explained how the (more equal) bargaining power between fishermen and dealers or traders emerges during the sorting performances:

“Often, almost always, when you meet with a fisherman to buy his catch, he will include ‘small’ fish in the bundle of ‘large’. We sort the fish together and when he tries to do that sometimes you complain but most of the times you better be quiet and let him do that because if you talk too much next time he will go sell to somebody else! So, you keep quiet and after you’re done with the transaction you have to sort the fish again. There are fishes in the ‘large’ bucket you know already will not be purchased by some shops or other dealers in Diani, so you take them out and put them in the ‘small’ bucket for the Mama Karanga to buy. You lose a lot of money like that but that’s the way it is”.

More equal negotiating power between local actors, short-haul distribution networks, and poor storage facilities, skew the categorisation of fish towards the category of fish-as-gift (and food), *kitoweo,* giving more agency to local people to access fish. The overall management of fish in the area is often couched in a language that makes repeated references to the household, the nurturing and feeding of family rather than fish as commodity, which as will be seen below is much more pronounced in Watamu. The categorisation as *kitoweo* is directly associated to the question of access—interviews with women on access to fish in the household revealed that in Shimoni availability and affordability of fish for the household is dependent on the (unpredictability of) conditions of the oceans, rather than commoditisation processes or the structure of the market. The most eloquent way in which this categorisation of fish in Shimoni emerged, and how that intersects the specific local conditions around access came from a woman interviewed in her double role as household head and (former) trader – as she put it:

“Availability of fish for our homes here in Shimoni depends on the ocean. If the ocean gives us fish today people are going to eat fish. If the ocean is stingy tomorrow, we won’t get fish tomorrow”.

Another woman (also a former trader) in Wasini island confirmed that either through dealers or fishermen she would normally get fish, pointing to the agency that local people can exercise:

“Fish availability here is very unpredictable; one day you get no fish another day you get a lot of fish; but even when the catch is small before taking fish to Shimoni fishermen would normally sell some of the catch here in Wasini for a cheaper price to maintain good connections with people; because the day they get a lot of fish, where are they going to sell it without fridges?”

Then elaborating on the relationships fishermen have with the local population:

“If you treat the villagers well, they will help you when you are in need! We are all related as family here in Wasini; fish is for our homes; you can’t deny people their *kitoweo*!”

### ‘Small’ fish, ‘large’ fish in Watamu

#### Market structure, spatial arrangements, marginalisation

Market structure and relations in Watamu differ from those in Shimoni in many different respects. In the first place, trolling, which is a method of fishing that involves dragging multiple fishing lines from a moving motorised boat, is a widespread technique and results in large catches both in terms of fish size and the overall biomass of the catch. Trolling is used to catch mostly large oceanic pelagic fish such as tuna, as the technique enables the exploitation of open waters (Abubakar et al. 2023). Trolling is a commercial activity that supplies higher-end national markets as well as the tourism facilities such as the many hotels and resorts of Watamu and surrounding locations given the much more developed tourist infrastructures (Kadagi et al. 2020).

Commercial fisheries of this kind dominate the BMU unit where 4 × 4 cars and motorised dealers and traders come daily to collect their catches. The improved infrastructure associated with tourism activities has resulted in a large capacity for storing fish which can preserve the cold chain in Watamu, enabling trade over longer periods of time, of larger quantities, and destined for distant markets. Watamu is known as a ‘hotspot’ for recreational and sport fishing (Kadagi et al. 2020). Although high membership fees mean these recreational and sports fishing centres are exclusive (Kadagi et al. 2021), local fishermen have also benefited in terms of fishing-related knowledge, gear, and equipment donations (interview with BMU member, Watamu).

Alongside trolling, more artisanal gears such as basket traps and handlines of the kind used in Shimoni are also used in Watamu. These kinds of fishing are done predominantly by migrant fishermen from Tanzania, unlike Shimoni where both local and migrant fishermen rely on this type of gear. In Watamu, fishing activities by migrant fishermen is seasonal, and targets the reef associated fish generally categorised as ‘small’ fish. This fishery avails itself of the in-depth knowledge of migrant fishers in targeting potentially higher concentration of fish (e.g. in proximity to conservation areas, coralline islands, and offshore reefs) (Wanyonyi et al. 2018).

This fishery is crucial when it comes to supplying more affordable ‘small’ fish for a broader spectrum of the population. These ‘small’ fish come from migrant fishers and are traded within the informal networks. They are cheaper, require quick sale (for not being able to be stored) (Darling 2014) and as a result are more likely to reach people’s plates locally. However, this fishery is faced with a number of constraints given the widening power gap in terms of resources at the disposal of actors operating at the BMU. Looking at the context-specific grading system of ‘large’ and ‘small’ fish again, is a useful lens to analyse questions of agency in terms of the capacity to access fish by the local population. 

The most evident form of vulnerability and marginality within the small-scale reef-associated fisheries is the result of a physical separation of the smaller-scale reef-associated local actors (fishermen and traders) activities from those of the BMU. This separation reflects the categorisation of ‘small’ and ‘large’ fish. While ‘large’ fish caught through more modern gear is handled (weighed, traded) at the BMU, another much smaller and informal site a few kilometres away has emerged called Darakasi, where smaller-scale dealers, traders and immigrant fishermen fish and trade in ‘small’ fish.

Darakasi is not formally recognised and fishing activities there occur only during the high season when migrant fishermen are around. The site emerged in response to the increasing marginalisation of small-scale actors who were considered to be posing an obstacle to the larger-scale fisheries operations at the BMU site, just few kilometres away. A small-scale male trader interviewed at Darakasi recalled these processes; he had operated at the site for a few years and remembered the tensions and high economic stakes at the BMU, whereby large-scale actors were securing access to financial support and better gears, ultimately leading to the separation of the two types of fisheries. Questions around concentration of resources and battles for power over external and government aid emerged:

“When I arrived in Watamu to do business all the aid was going in the hand of few people at the BMU, while mamas were never welcome. Some people were getting richer and richer, those who were close to the BMU and were getting the best gear from outside. The smaller guys, traders and fishermen received no aid and no support – sometimes they were prevented from conducting their activities at the BMU”.

#### From marginalisation to adaptation: local arrangements, opportunities, and constraints for reef fisheries

Despite the marginalisation of actors operating at the Darakasi site, the informal character has the advantage of providing a platform for poorer actors to conduct their business. It facilitates the continuation of a vital fishery that enables local people to access fish through local circles of markets and exchange (Darling 2014). More casual relationships and less stiff competition exist at Darakasi as compared to the formal site of the Watamu BMU where high economic stakes often lead to conflict and arguments among fishermen and traders.

As the same trader quoted above argued, the emergence of the Darakasi site brought positive change and more opportunities for marginalised actors:

“I tried to do business at the BMU but received no support because I didn’t have enough money to buy large catches from trolling – I knew that mamas had been doing some trading of ‘small’ fish at the Darakasi site, so I joined some other traders and we decided to shift our activities there. When we shifted to Darakasi, mamas were very happy because more fishermen came along when they knew that we wanted to buy their fish. Today we have quite a few fishermen from Tanzania who come here and sell fish to us. I and other traders buy small fish for my shops and mamas buy smaller fish for their market”.

At Darakasi, sorting arrangements are made to ensure a fair allocation of fish from the catch. Weighing and sorting fish occur smoothly, without conflict or arguments or people scrambling for the fish – instead Mama Karanga line up their baskets while fish is weighed, waiting for it to be allocated. Normally an external person, a dealer or fisherman, volunteers to arrange fish in bundles of 4–5 fish on the ground—they would use their skills to ensure that all bundles have a similar value. Trading off size for quality is common, that is, a bundle that includes some fish of visibly lower quality would compensate with more valuable fish of a bigger size and better appearance. The quality of fish is therefore judged visually by the person sorting the fish according to features such as how the eyes of the fish look (pale whitish eyes indicate low quality while clear shiny eyes indicate fresh, high quality fish), whether the fish has abrasions or injuries, redness of the gills (pale brownish and dark gills indicate low quality and fresh red gills high quality), and/or whether the fish is characterised by many tiny bones within the flesh (less bones in flesh associated with high quality fish).

Most people consulted at the site expressed overall satisfaction with the social and business relationships between different actors which allow them to supply local markets with affordable fish for the local population However, they also voiced dissatisfaction about the material and institutional conditions in which they conduct their business. Apart from the poor physical conditions, lacking equipment and space dedicated to handling and processing fish, the status most artisanal fishermen share at the Darakasi site, is as ‘immigrants’ which does not facilitate a sustained and reliable supply of fish throughout the year.

Informal discussions with a range of stakeholders in Watamu (BMU members, fisheries officers, dealers, and traders) on the topic of immigrant fishermen brought out mixed feelings. Although, immigrant fishermen’s in-depth fishing knowledge and skills were acknowledged by local fishermen, a number of negative aspects of their presence were also mentioned, in particular an increase in the competition for fish and the lowering of the price of fish, due to migrant fishermen ‘flooding’ the market with (cheaper) fish (Crona et al. 2010). The immigration status of the ‘migrant’ fishermen is unclear, leaving them to conduct their fishing activities within a policy ‘vacuum’ (Crona et al. 2010) which adds to their marginality. The lack of clarity on the immigration status of these fishermen adds to the seasonality of their activities in Kenya which in turn negatively affects the availability of affordable fish (during the low season) and consequently affects people’s agency to access fish locally. One migrant fisher from the Tanzanian island of Pemba, conducting his activity at the Darakasi site, explained how his unclear migration status tends to shorten and minimise his fishing activity in the country:

“I have been coming here for around five years, but it’s difficult. I have to take into account that something can happen, other local fishermen can tell me to leave, or the authorities can arrest me. Sometimes when we feel that the situation can degenerate, we just leave before the end of the season. That is too bad because we lose a lot of money, and all the traders we supply, but that’s better than being jailed because when you are jailed you have to pay fine after fine and that eats away all the profit you’ve made”.

On the one hand, the rise of the Darakasi site is an expression of local organisational capacity in adapting to resource scarcity (Cinner et al. 2015), fulfilling also a nutrition-related function in supplying affordable ‘small’ fish for the poorer consumers in Watamu. On the other hand, it is the result of marginalisation and unfavourable factors that need to be accounted for and acted on in the arena of fisheries governance.

### Fisheries governance and food: acting on markets and institutional structure

At the workshop organised in Shimoni to validate findings from fieldwork, two major areas around local governance emerged as crucial when it comes to making governance more sensitive to the needs of the local population for food; these were the role and potential of trade conducted by women small-scale traders, and the necessity to strengthen the role and capacity of the BMUs in mediating between actors. These are two areas that are closely connected (Matsue et al. 2014; Nunan & Cepić 2020); the functioning of BMUs as catalysts of participation in fisheries governance in Kenyan fisheries, either inland or coastal, has been marred by a number of conflicts over resources (Murunga et al. 2021), unsustainable fishing practices (Etiegni et al. 2017), and poor integration of formal and informal institutions and rules, with the latter often overriding the first, undermining compliance with rules that BMUs are supposed to implement on the ground (Etiegni et al. 2020). When it comes to women’s participation in co-management through formal structures (i.e. BMUs), despite women’s presence in committees (Nunan & Cepić 2020), financial and social barriers such as lack of assets and unbalanced gender power relations have posed obstacles to effective participation (Matsue et al. 2014).

These challenges around women’s participation were highlighted at the stakeholder workshops with remarks on the (im)morality of market relations, and management capacity in connection to the persisting marginal role of women traders. One Shimoni BMU executive expressed his opinion on the overall local management capacity in terms of making markets fair for all actors:

“We don’t know how to manage our markets. Our markets are not fair - we give priority to dealers because of the price they can offer rather than helping our own mamas do their business. We know that dealers can give us a better price so we sell fish to them to nurture the relationships and forget our mamas! This is not good management”.

The role that Mama Karanga had during the Covid-19 crisis emerged as crucial in buffering the losses incurred by fishermen and dealers. As businesses shut and movements were restricted the only markets and trading that went on were local trading for household consumption. Mama Karanga became crucial as suppliers of cheap fish that the financially struggling local communities could afford. As a way of sensitizing the audience at the workshop on the necessity for more inclusive markets, one of the KMFRI^4^ staff highlighted:

“Let us remember our mamas; don’t you all remember what happened just some time ago when we were hit by the covid wave? The dealers started calling mamas, almost begging them to buy the fish because they didn’t have anywhere else to sell it! Now that luckily the crisis is behind us they are back to their struggling situation, battling with high prices as usual. But you need to work together to change this situation”.

Comments on the key role of Mama Karanga stirred up reactions by workshop participants who showed agreement and support. This further led to a discussion on the relationships between BMU executives and dealers. Several BMU members from all three BMUs invited (Shimoni, Watamu, Uyombo) identified the lack of or poor services and facilities offered by the BMUs to dealers negatively impact the BMU executives’ ability to act on market relations and advocate for dealers to sell more fish (and more affordable) to women traders. One member of the Uyombo BMU argued:

“In our BMU we have no facilities or services to offer to our dealers, beyond weighing the fish for them; we only have one scale – no storage facilities, fridge, fishing equipment. So, you see, this prevents us from developing good relationships of mutual help with the dealers, and when they favour selling their fish for higher end markets, we have no say, we can’t ask them to give up profit with nothing in return”.

Efforts at sensitisation are made to encourage dealers to support women’s trading activities by selling more fish to them, despite the poor conditions around the services provided by the BMUs. In a post-workshop follow-up discussion to validate findings, one county officer mentioned that BMUs and counties seek to sensitise dealers on the benefits for the local communities from selling more fish to Mama Karanga – however, this sort of sensitisation is limited to personal initiatives, and is not part and parcel of the institutional structure:

“At the BMU sometimes we try to do some sensitisation among the dealers and fishermen to make sure that mamas get their own share of fish; we tell them to sell more fish to mamas. Sometimes they listen, so they set aside more fish for them, especially the small fish and the discards – but we know that is not enough. Until something is done at the level of local government, I think Mama Karanga will continue to struggle”.

## Conclusion

The ethnography in this article has revealed local fish categorisations that impact people’s agency in accessing nutritious fish, particularly for the sectors of communities who are living in poverty. We have analysed these market dynamics through the lens of performativity (Mather et al. 2017) which allowed us to deconstruct existing management categories, based on size and species, to ‘enact’ new classifications of fish based on local values and knowledge (Lien & Law 2011). These processes of classification of fish determine how fish enters or is kept outside of circles of commoditisation in the market. Power and agency are at the foundation of these processes (Cardwell 2015) eventually determining whether fish reach (or not) people’s plates.

Scarce resources (financial) and equipment (gear, storage facilities) are related to better access to fish by local communities in Shimoni where much of the catch ends up in the basket of ‘small’ fish and is likely to enter local rather than more distant markets. On the contrary, better resources and gear in Watamu have led to the marginalisation of actors involved in the fishing and trading of ‘small’ fish with negative consequences on access to fish as food by the local community. The comparison problematises assumptions in food-related policy that more food (ie. improved production) has direct spillover effects on the food and nutrition security of the poor and vulnerable (Foilleaux et al. 2017). Hence, the necessity emerges to introduce understandings of power and agency (Ibrahim & Alkire 2007) into fisheries management to make fish more affordable and accessible for those who need it most.

This paper adds important insights to the understanding of current transformations in territorial markets (Bavinck et al. 2023) by adding further layers of analysis at a more localised scale to the debate around trade-offs between revenues from trade and food security (Asche et al. 2015). Being aware of how local categories of fish come about within surrounding development and market-related conditions is particularly relevant given the growing interest in the role of ‘small’ fish for people living in poverty (Bavinck et al. 2023; Schut & Weeratunge 2024). Departing from a size-based trait of all fish under 25 cm, ‘small’ fish is currently defined not simply by its size but by characteristics and values around its affordability and practices of processing and consumption, in order to bring out its (undervalued) role for food and nutrition security among low-income populations (Bavinck et al. 2023). Here, we have brought out the processes through which ‘small’ fish is constructed in opposition to ‘large’ fish through a relational perspective; beyond size, what fish is deemed ‘small’ depends on localised relations of power, which in turn are determined by broader socio-economic, political, and material circumstances.

Through the relational perspective, this article improves our understanding of the role of actors involved in small fish value chains, the values underlying their practices, and those assigned to (small) fish itself. Here, we have shown how all these practices and values combined contribute to either enable ‘small’ fish value chains to cater to low-income populations or restrict the spaces in which ‘small’ fish value chain actors can operate. In doing so, we endorse the need to move from a focus limited to economic dimensions of the added (economic) value of fish as it moves from harvest to consumers, to a broader perspective that focuses on social and political dimensions determining access to (small) fish by local populations (Bavinck et al. 2023; Schut & Weeratunge 2024). Small fish value chains for nutrition remain a blind spot in Kenya as in most of the Global South from Africa to Asia (Schut & Weeratunge 2024), given the blue economy narratives and policies favouring increased production over the food-related benefits from consumption; this article intends to contribute to a move towards nutrition-sensitive management of fisheries that can reconcile profit motives with food-related objectives.

## Data Availability

The data that support the findings of this study are available from the corresponding author upon request.

## Footnotes

1 Fish names reported in this paper are broad taxa categories that correspond to the respondents’ ways of calling or identifying fish. Each taxa group constitutes several species that may not be exhaustive. We can only give examples of what are the dominant species in each category based on fisheries data and experience with catch assessments in coastal Kenya.

2 The ethnography and other fieldwork activities were conducted by the first author only.

3 The National Commission For Science, Technology & Innovation.

4 Kenya Marine and Fisheries Research Institute.
